# Clear Aligner Treatment of a Class II Division 2 Patient With Multiple Anterior Crowns and Molar Mesial Tipping Through Cantilever Arms: A Case Report

**DOI:** 10.1155/crid/5437508

**Published:** 2025-09-15

**Authors:** Yuetian Li, Zhouqiang Wu, Guannan Hu, Yan Wang, Hu Long, Wenli Lai

**Affiliations:** ^1^State Key Laboratory of Oral Diseases, Department of Orthodontics, West China Hospital of Stomatology, Sichuan University, Chengdu, Sichuan Province, China; ^2^Department of Orthodontics, Stomatology Hospital, School of Stomatology, Zhejiang University School of Medicine & Clinical Research Center for Oral Diseases of Zhejiang Province & Key Laboratory of Oral Biomedical Research of Zhejiang Province & Cancer Center of Zhejiang University, Zhejiang, Hangzhou Province, China

**Keywords:** anterior crowns, cantilever arms, Class II Division 2, clear aligner

## Abstract

Patients with Class II Division 2 malocclusion, often presenting with severe deep overbite and poor facial esthetics, are one of the most challenging cases in orthodontic practice. This case report shows the successful treatment of a Class II Division 2 deep bite patient with multiple anterior crowns using clear aligners. After the extraction of Tooth 45, we moved the right mandibular molars mesially to achieve a Class I relationship. Cantilever arms and elastics were used to correct the molar mesial tipping. We precisely controlled the space between prostheses by digital design. At the end of the treatment, new porcelain crowns and the counterclockwise mandibular rotation improved the dental and facial esthetics.

## 1. Introduction

Class II patients with deep overbite always have a retrognathic mandible, resulting in a compromised facial profile [1, 2]. In treating this kind of patient, orthodontists always perform intrusion of anterior segments with miniscrews as well as posterior extrusion to open the deep bite [3, 4]. However, the extrusion of posterior teeth can cause clockwise rotation of the mandible, which is detrimental to the profile improvement. Therefore, vertical control is necessary in deep bite correction.

Traditionally, orthodontists used fixed appliances and miniscrews to treat Class II patients with deep bites. However, maxillary and mandibular dentition must be treated in sequence since brackets cannot be bonded to the lower teeth until the deep bite opens. Besides, bonding the bracket on the prosthesis is difficult and caducous. Recently, the usage of clear aligners treats both arches at the same time and avoids the bond of brackets on the prosthesis, which shortens the treatment time [5, 6]. However, the use of clear aligners is controversial [7]. On one hand, it meets the esthetic demand of patients and is beneficial to dental health [8–10]. On the other hand, some movements are difficult to achieve, including extrusion and torque control, especially in complex malocclusion cases [11, 12]. Although it remains controversial in the effectiveness of treating severe malocclusions, partially because some of the tooth movements may not be predictable enough, many researchers have reported successful cases to prove that the clear aligners today have been able to treat mild to severe malocclusions [13, 14]. The occurrence of new tools such as precision cuts and button cutouts allows the use of intermaxillary traction, which significantly increases the success rate of treatment [15]. Thus, every case is hopefully a clear aligner case. In summary, clear aligner has become a significant esthetic alternative in orthodontic practices.

This case report describes the efficient treatment of a Class II patient with deep overbite, multiple crowns, and temporomandibular disorder (TMD). The total treatment period was 18 months. Invisalign clear aligners (Align Technology Inc., Santa Clara, California) were used to control the occlusal plane, arrange the space for prosthodontics, and correct the molar relationship.

## 2. Diagnosis and Etiology

A healthy female patient aged 25 visited the hospital to receive treatment for her deep bite, and clear aligners were strongly demanded by this patient because of her occupation. Her medical history was nonspecific.

**Figure 1:**
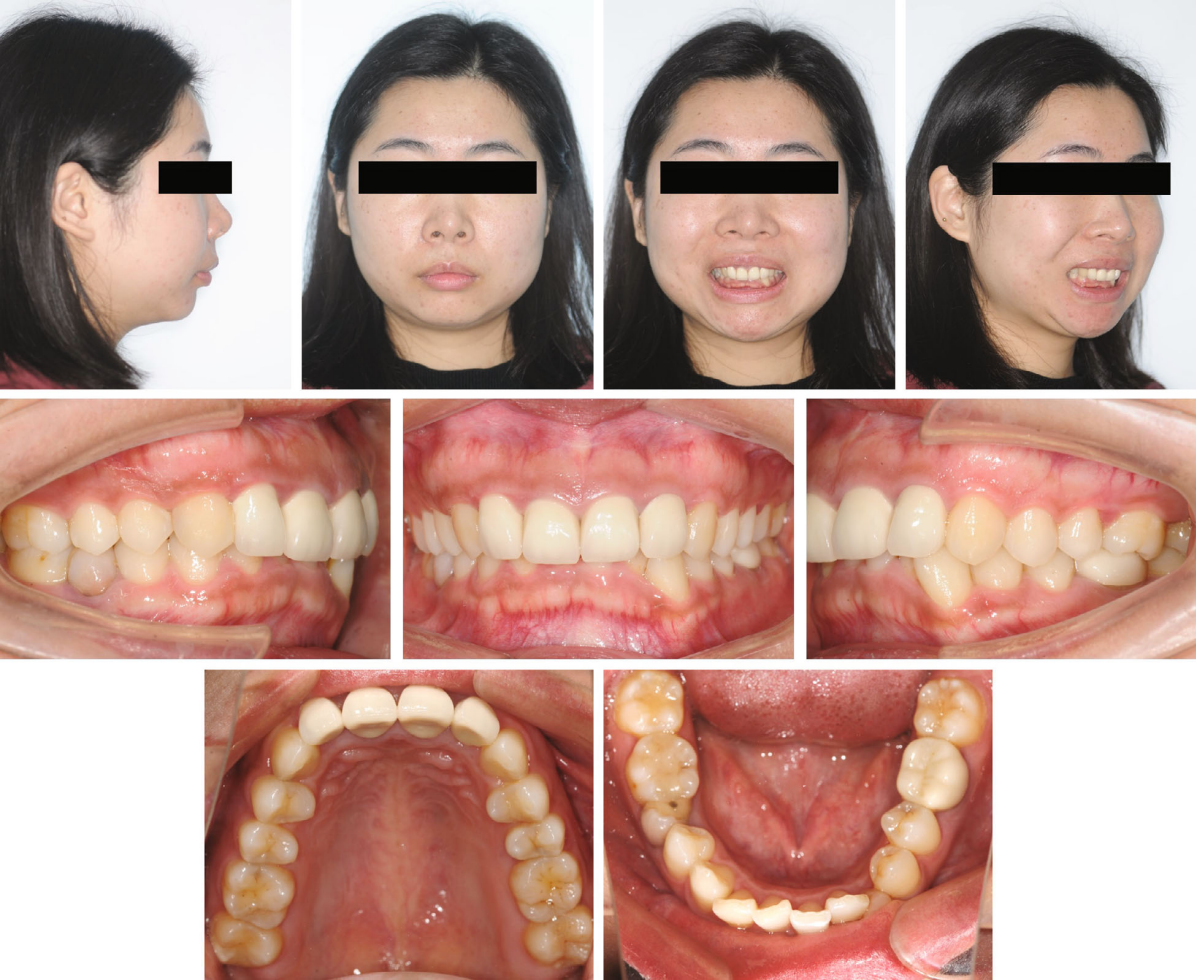
Pretreatment facial and intraoral photographs.

**Figure 2:**
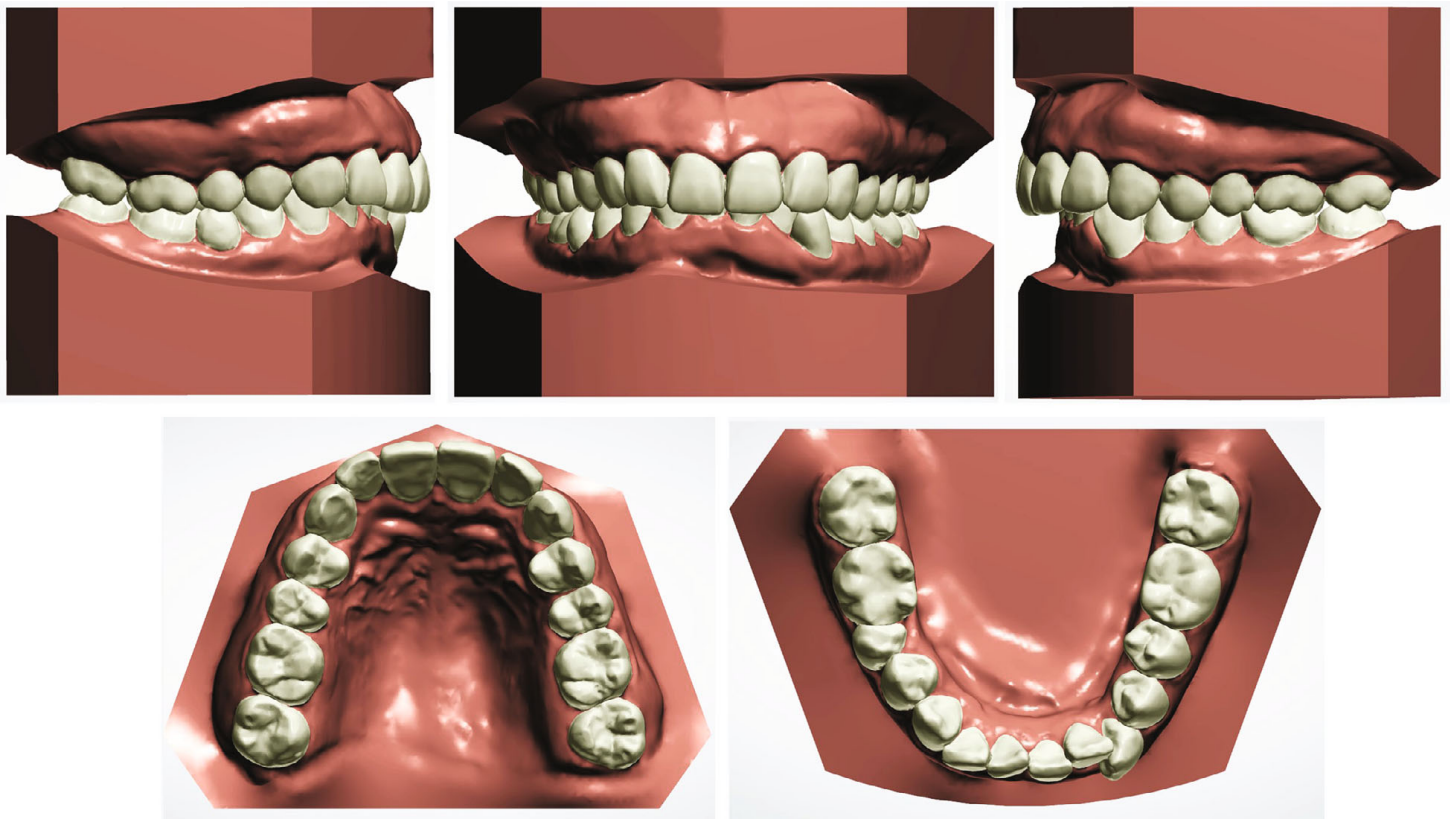
Pretreatment digital study models.

Clinical examination revealed a bilateral click during mouth opening. There was no pain or limitations when evaluating other temporomandibular joint (TMJ) symptoms. The facial photographs (Figure 1) exhibited a protrusive profile with an incompetent lower lip. The intraoral examinations (Figure 1) and the analysis of the digital study models (Figure 2) showed a large overjet (5 mm), deep overbite (7 mm), moderate dentition crowding of the lower arch, and an anterior Bolton ratio of 84%, which meant the width of the lower anterior teeth was wider than normal. The right molar relationship was Class II, while the left was Class I. Teeth 12–22 and 36 were porcelain fused to metal teeth (PFM), and the crown of Tooth 45 showed discoloration and inclined distally. Dental midlines were centered with the facial midline. There was no other specific dental or medical history.

**Figure 3:**
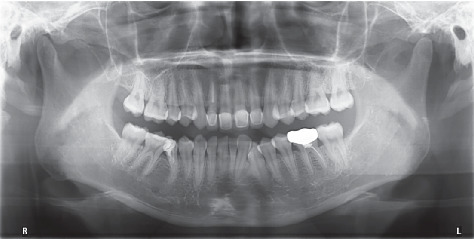
Pretreatment panoramic radiograph.

Pretreatment panoramic radiograph (Figure 3) exhibited that Teeth 12, 36, and 45 had received incomplete root canal treatment. All of the third molars had been extracted. The shape of the bilateral condylar morphology of the patient was normal. The alveolar bone of both arches showed moderate horizontal resorption.

**Figure 4:**
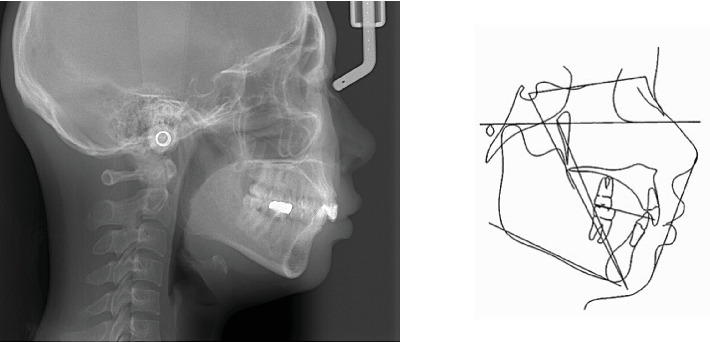
Pretreatment lateral cephalometric radiograph and tracing.

Pretreatment lateral cephalometric radiograph (Figure 4) in maximum intercuspation exhibited a protrusive profile. The cephalometric measurements (Table 1) indicated a normal vertical skeletal pattern (Frankfort mandibular plane angle [FMA]: 26.4°). However, the sagittal pattern was Class II (ANB: 7.5°) due to the mandibular retrognathia (SNA: 83.2°, SNB: 75.7°). The lingual inclination of the upper incisor (U1-SN: 91.6°) and the lower incisor (L1-MP: 93.6°) was observed. The soft tissue was normal (upper lip to esthetic line: 2.1 mm; lower lip to esthetic line: 4.2 mm).

The patient's diagnosis was TMD, Angle Class II Division 2, skeletal Class II malocclusion, mandibular retrognathism, and deep overbite.

### 2.1. Treatment Objectives

The chief complaint of this patient was to improve deep bite. The primary objective was to correct the deep overbite and large overjet without mandible clockwise rotation. The additional objectives were to establish a Class I molar relationship, eliminate dental crowding, and improve her incompetent lower lip without aggravating TMJ burden.

### 2.2. Treatment Alternatives

Double-jaw surgery including maxillary LeFort I recession and mandibular bilateral sagittal split ramus osteotomy (BSSRO) (advancement genioplasty if necessary) is a possible treatment option due to sagittal skeletal discrepancy [16]. However, the patient did not want a surgical intervention. Traditionally, using fixed appliances and miniscrews is another choice. However, it was denied at the beginning as the Teeth 12–22 and Tooth 46 of the patient are porcelain fused to metal crowns.

To improve the Class II molar relationship, there are two common nonsurgical treatment methods. One way is extracting two maxillary premolars to acquire a complete Class II molar relationship. Another way is maxillary molar distalization or uprighting [17]. This patient was 25 years old with a unilateral molar relationship discrepancy and a normal maxillary base bone with a normal nasolabial angle. As a result, the extraction of the maxillary premolars was inappropriate for her. In this case, with discoloration and distal inclination of Tooth 45, maxillary molar uprighting and unilateral mandibular premolar extraction were performed on the patient to improve the molar relationship.

To improve the incompetent lip, due to the short anterior facial height and the axis of the incisors, the lingual inclination of the upper incisors should be corrected. Interproximal enamel stripping of the prosthesis of the upper incisors and the uprighting of the molar provided the space for the change of the axis of the upper incisors. Meanwhile, the intrusion of lower incisors was performed. The counterclockwise rotation of the mandible and forward positioning of the chin improved the protruding profile and the incompetent lower lip [18, 19]. Besides, the occlusion plane's counterclockwise change helped to improve the incompetent lower lip.

As Teeth 12–22 and Tooth 46 of the patient are porcelain fused to metal crowns, it was difficult to bond brackets. Therefore, it is complicated for the patient to use traditional straight wire appliances. In addition, patients have a strong desire to use clear aligners, so we finally chose the Invisalign clear aligner appliance. After a discussion with the patient, the extraction of Tooth 45 and the alignment of the upper midline to the midline of Tooth 31 were finally planned.

### 2.3. Treatment Progress

**Figure 5:**
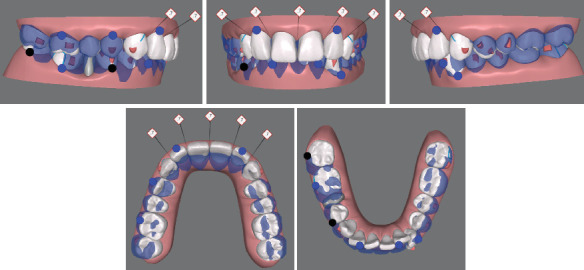
ClinCheck plan of the first stage.

In the first stage (Figure 5), 38 aligners in total were designed. We also designed interproximal enamel stripping of the upper incisors to divide the upper anterior prosthesis and to optimize the Bolton ratio. Then, distalization, uprighting, and intrusion were designed for bilateral maxillary molars as well as protrusion of anterior upper incisors. For the mandible, we extracted Tooth 45 at the beginning. Bilateral buttons were placed on the buccal surface of Teeth 37 and 46, preparing for Class II traction after the completeness of the uprighting of the maxillary molars. Class II traction was designed to avoid the extra labial inclination of Teeth 12–22 as well as help Teeth 46 and 47 move mesially. After the mandible right molars' movement was completed, the lower incisors were designed to be intruded and moved to the right.

**Figure 6:**
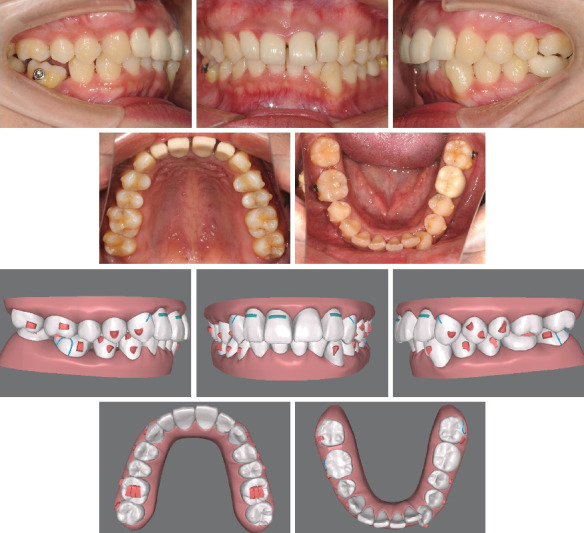
Intraoral photographs and ClinCheck plan of the #22 step.

**Figure 7:**
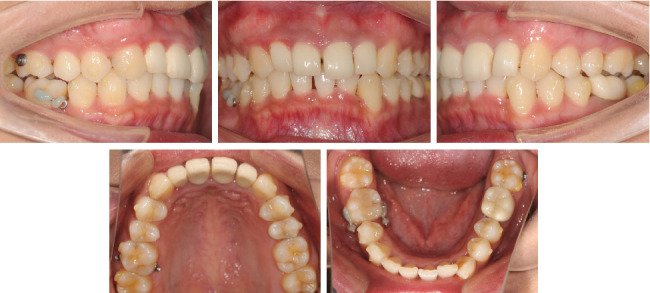
Intraoral photographs of the #32 step.

In the eighth month of treatment at the #22 step (Figure 6), during mesial movement of the molars, we found that Tooth 46 inclined mesially compared to the ClinCheck plan. To solve this problem, we replaced the button with power arms (both the buccal and lingual side of Tooth 46) to correct the inclination with elastic to Tooth 16. After 4 months of traction at the #32 step (Figure 7), the effect was obvious.

**Figure 8:**
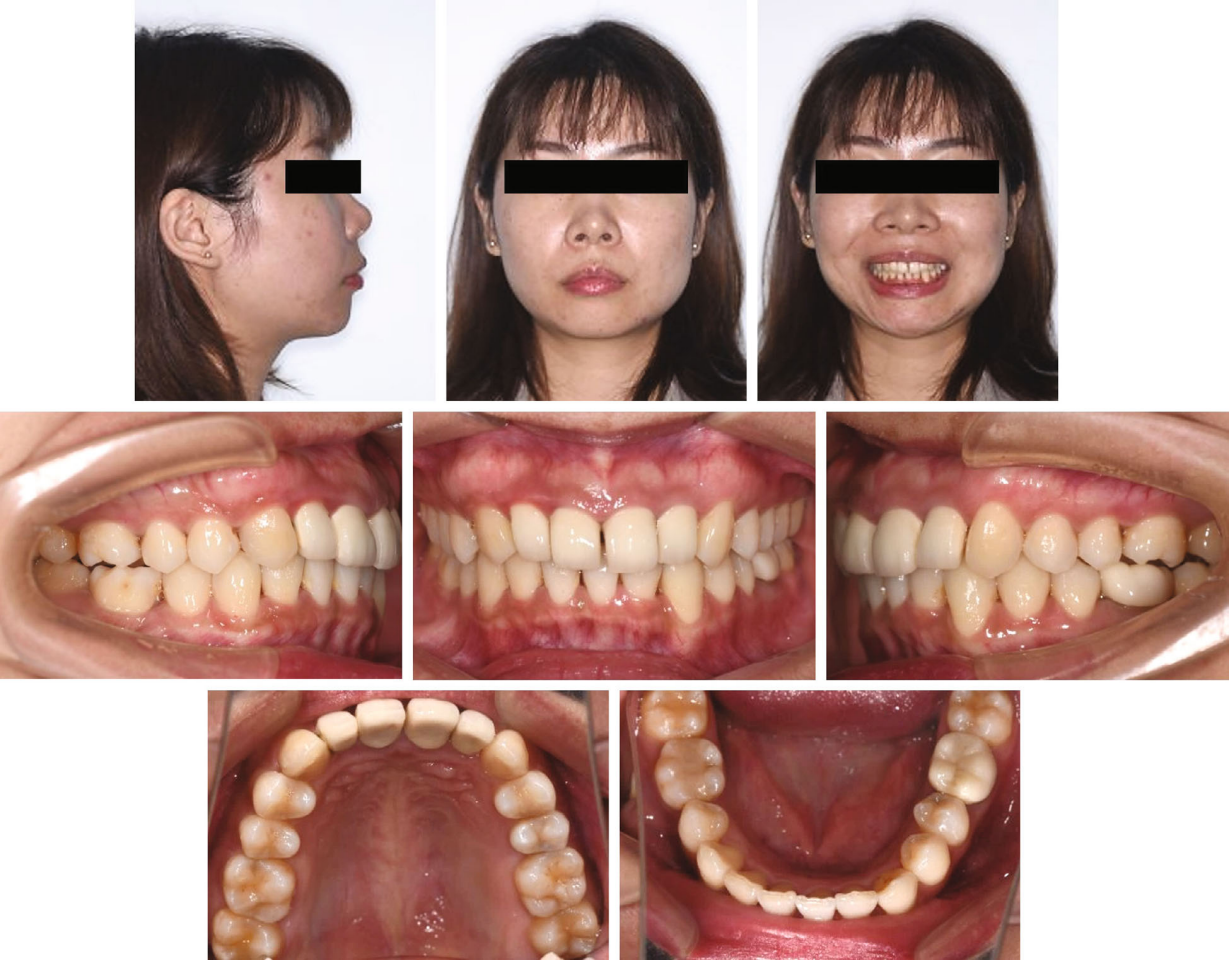
Facial and intraoral photographs when starting the refinement phase.

**Figure 9:**
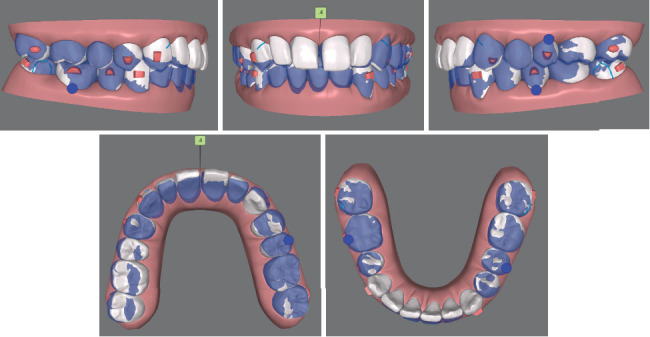
ClinCheck plan of the refinement stage.

**Figure 10:**
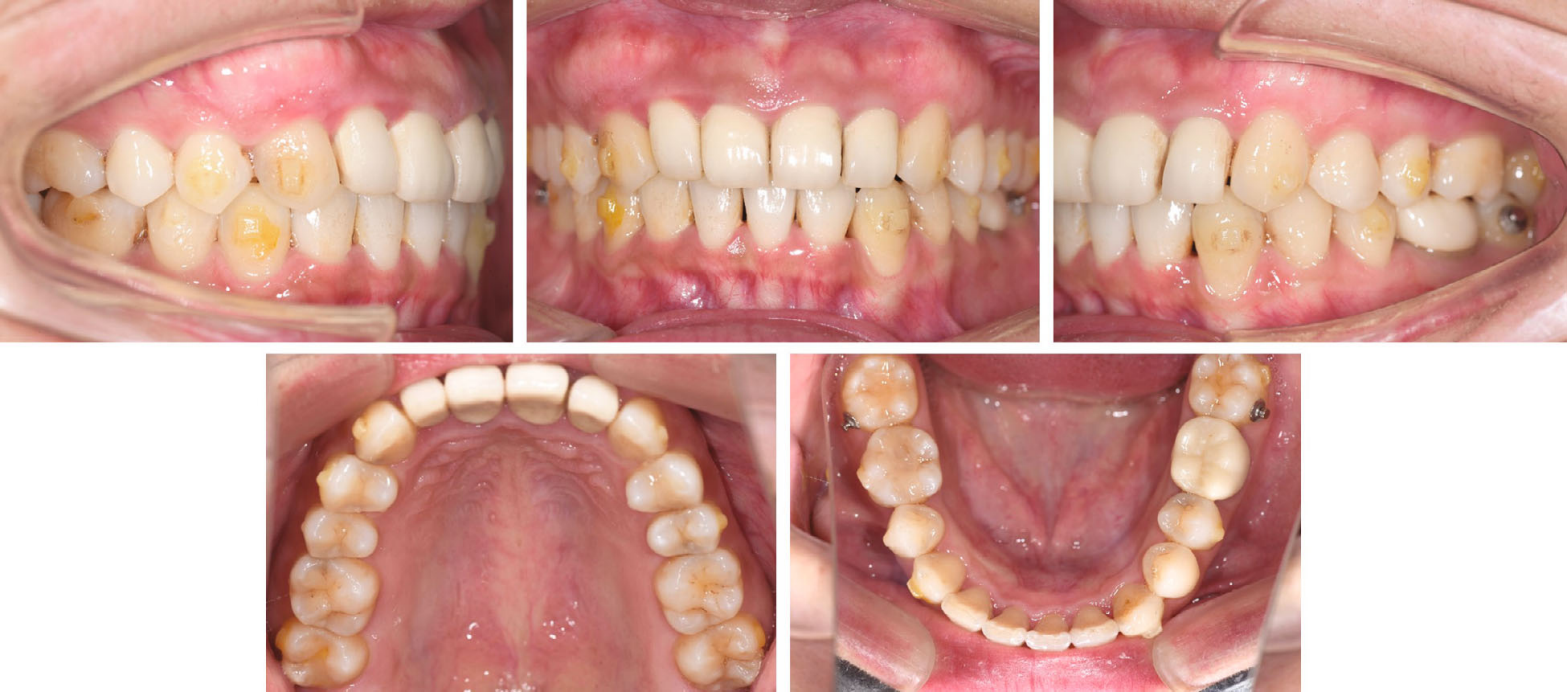
Intraoral photographs of the AA#15 step.

After 1 year of treatment, we started the refinement phase to achieve a better occlusion (Figure 8). In this phase (Figure 9), we also designed the uprighting of the maxillary molars and the intrusion of the lower incisors, aiming to achieve the maximal degree of counterclockwise rotation of the mandible. Fifteen aligners were designed in this phase, and the patient cooperated well (Figure 10). After the orthodontic treatment, all multiple porcelain fused to metal crowns of Teeth 12–22 and 36 were remade.

During all stages, the aligners were changed every 10 days. All designed stages of the process should be fulfilled and should not be skipped. Every aligner should be worn 22 h a day, and the chewing stick should be used 20 min a day. As we all know, the effect of orthodontic treatment by clear aligners depends on the patient's compliance. In this case, the patient's good compliance resulted in a short treatment course. At the end of treatment, the clear thermoplastic retainers were used to stabilize the occlusion.

### 2.4. Treatment Results

**Figure 11:**
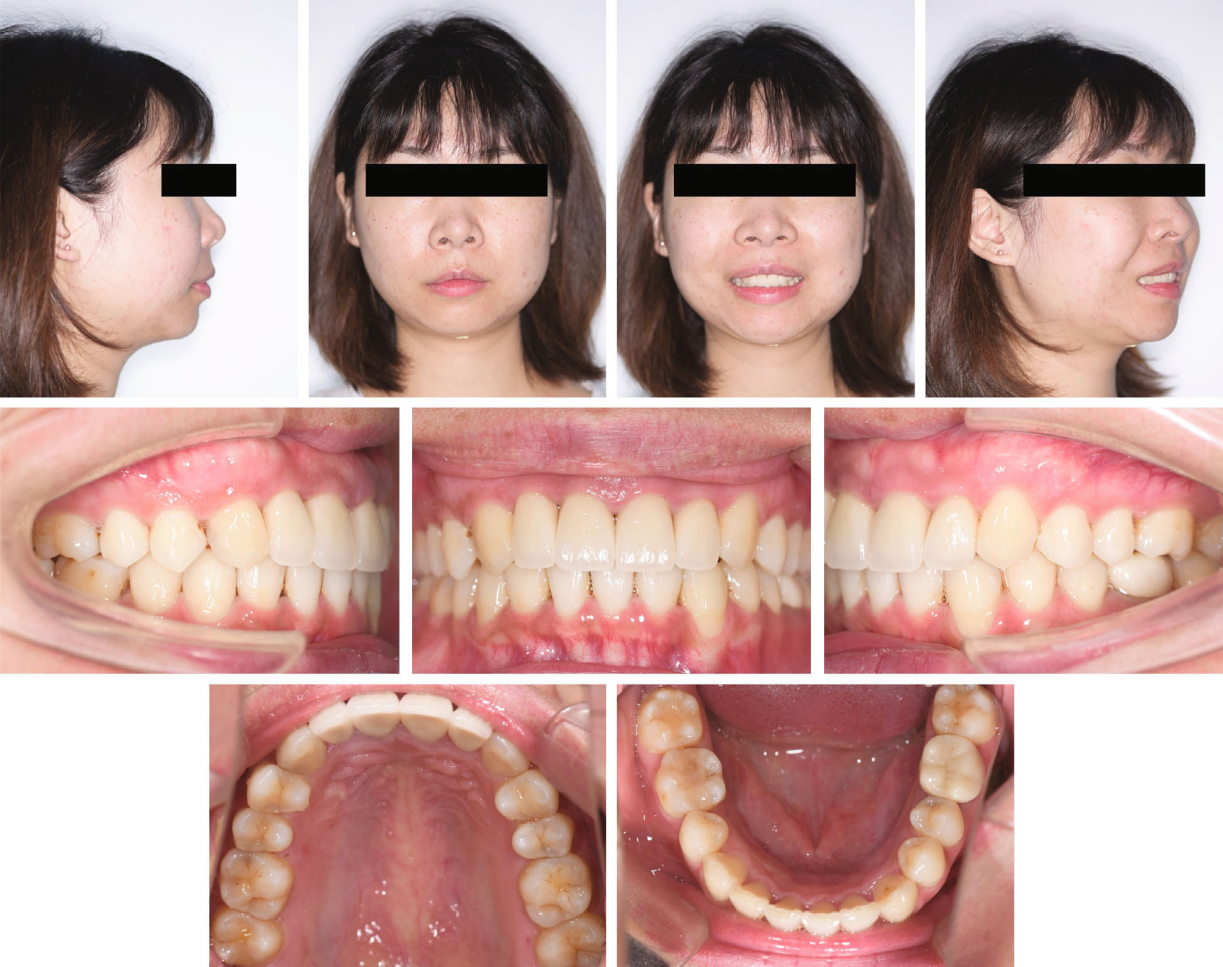
Posttreatment facial and intraoral photographs.

**Figure 12:**
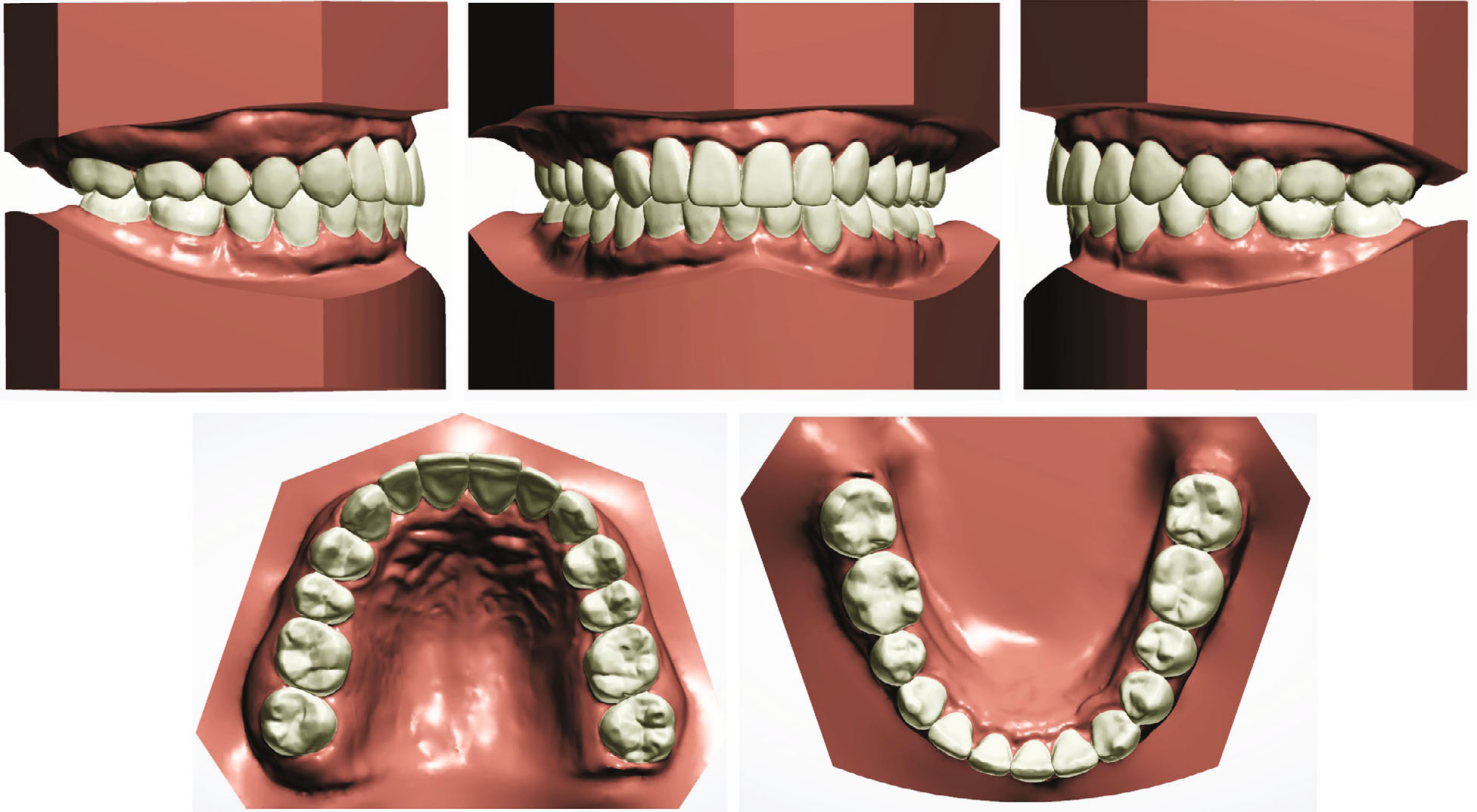
Posttreatment digital study models.

Intraoral photographs (Figure 11) and the digital study models (Figure 12) showed a bilateral Class I molar relationship, adequate overbite and overjet, well-aligned teeth, and maximal occlusal contacts that were reached. No gingival inflammation and noticeable white spot lesions were found. The dental midline of the upper arch was in line with the middle part of the lower incisor with an anterior Bolton ratio of 76%. Evaluation of TMJ symptoms showed no pain or limitation, and the click during mouth opening disappeared. Facial photographs (Figure 11) showed improvement in the facial profile, essentially because of adequate posterior teeth intrusion, which contributed to the counterclockwise rotation of the mandible and forward positioning of the chin.

**Figure 13:**
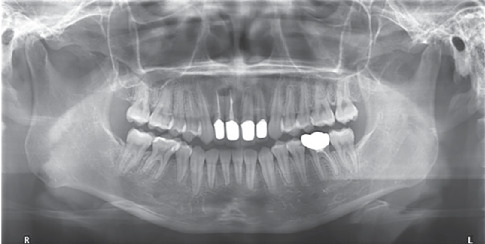
Posttreatment panoramic radiograph.

**Figure 14:**
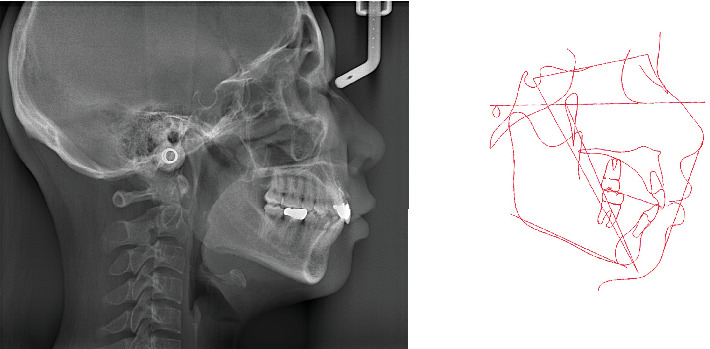
Posttreatment lateral cephalometric radiograph and tracing.

**Figure 15:**
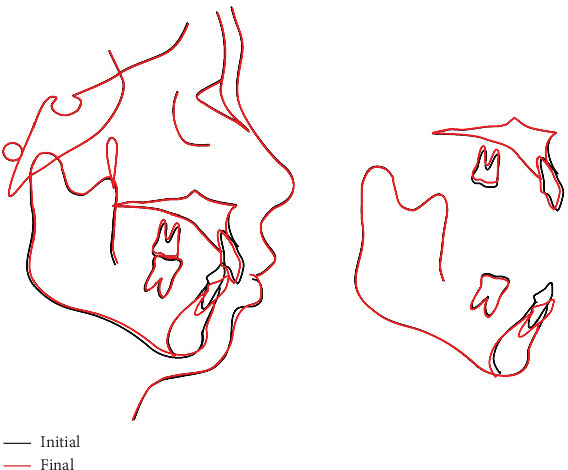
Superimposition of pretreatment and posttreatment cephalometric tracings.

The final panoramic radiograph (Figure 13) confirmed no root resorption, and the degree of parallelism between teeth roots was also improved. At posttreatment cephalometric analysis (Table 1 and Figure 14), it is possible to identify that SNB increased from 75.7°to 76.1°, ANB decreased from 7.5°to 6.2°, and both FMA and MP-SN also decreased, which demonstrated an improvement of the profile. According to the cephalometric tracing superimposition (Figure 15), the amount of molar intrusion was 1 mm, the amount of lower incisor intrusion (lower incisor to the mandibular plane) was 5.2 mm in mandibular superimposition, the Frankfort plane to mandibular incisor angle (FMIA) decreased 11.4°, and the amount of upper incisor intrusion was 2.2 mm. In summary, our treatment objectives have been basically achieved.

**Figure 16:**
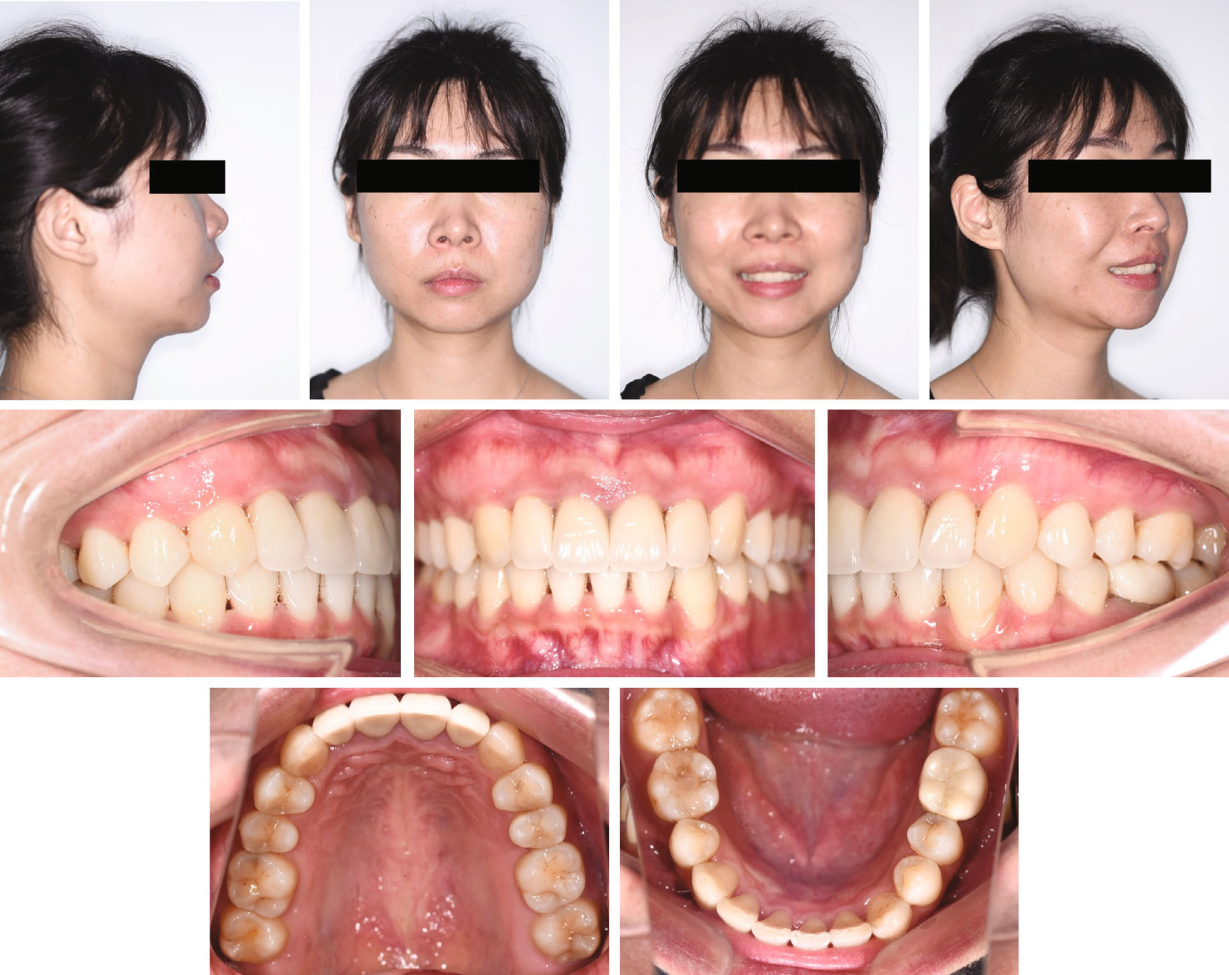
Facial and intraoral photographs after 15 months retention.

After 15 months of retention, the patient visited the hospital for a check-up (Figure 16). It is obvious that her occlusion was stable, and the consistency of the treatment was observed. The patient used and will continue to use clear thermoplastic retainers every day.

## 3. Discussion

**Figure 17:**
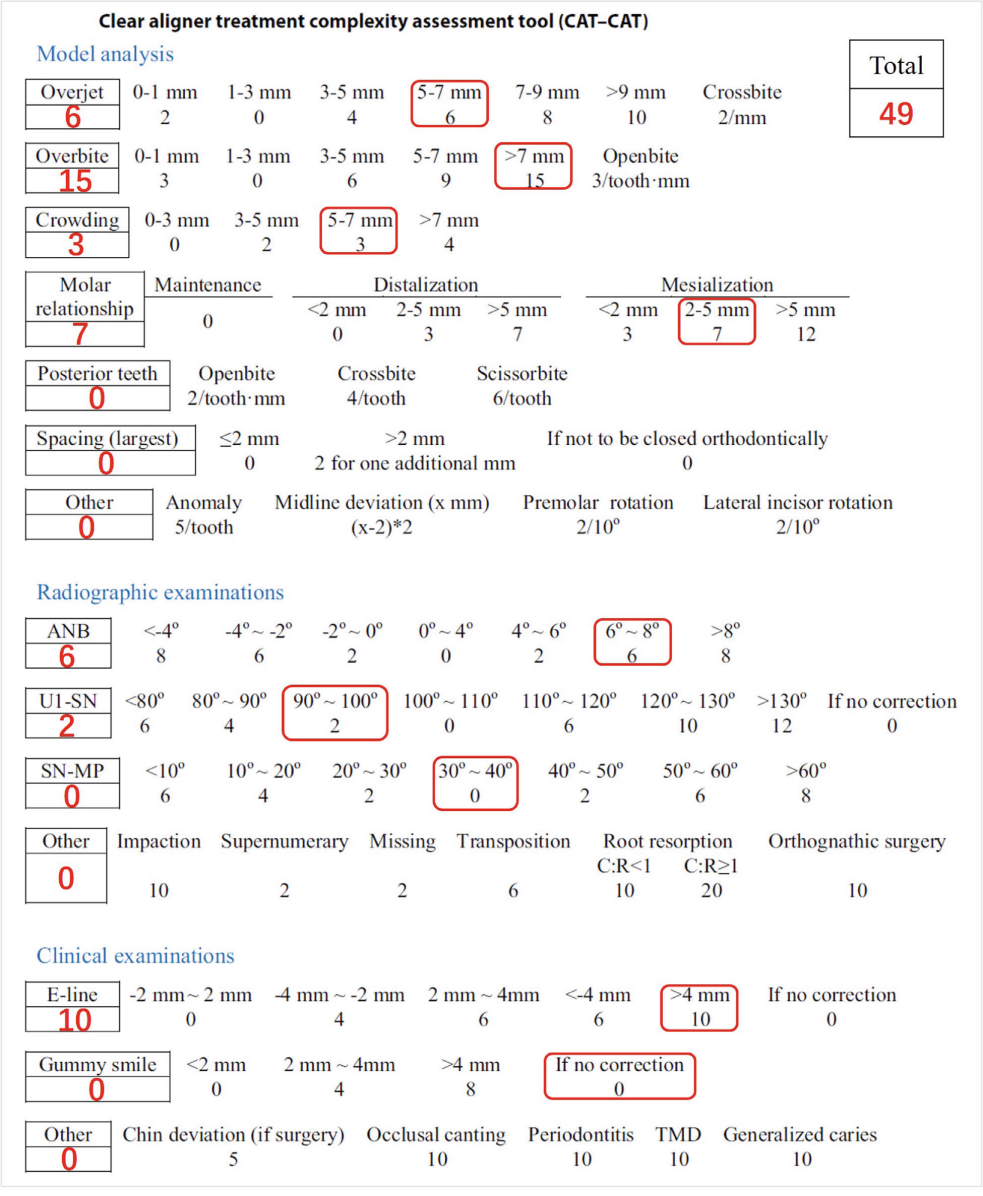
Score of clear aligner treatment complexity assessment tool (CAT–CAT).

Although this was a complex case according to the clear aligner treatment complexity assessment tool (CAT–CAT) (Figure 17) [14], we chose Invisalign clear aligners for the following reasons. As for the traditional fixed appliances, we needed to bond the maxillary bracket first to correct the axial direction of the upper anterior teeth of Class II Division 2 and then bond the mandibular bracket, which would prolong the treatment procedure. However, the clear aligners could correct both arches at the same time. Besides, because of multiple porcelain fused to metal prostheses, it was difficult to bond brackets on the surface. Clear aligners could avoid this problem by not designing attachments on specific teeth or using prostheses affinity with the resin. Furthermore, the patient's chin deficiency needed appropriate vertical control to avoid a compromised facial profile. Clear aligners did better in vertical control than fixed appliances. On the one hand, clear aligners could avoid molar extrusion because of the 1.5 mm thick material between the occlusal surfaces [20, 21]. On the other hand, they could ensure sufficient anchorage by designing special teeth moving patterns, such as the step-by-step intrusion pattern during anterior teeth intrusion and molar distalization or uprighting for reinforcing the anchorage [22]. Meanwhile, by designing the upper molar distalization, the Class II molar relationship would also be corrected. It is also convenient to use clear aligners to arrange the spaces for prosthodontics.

**Figure 18:**
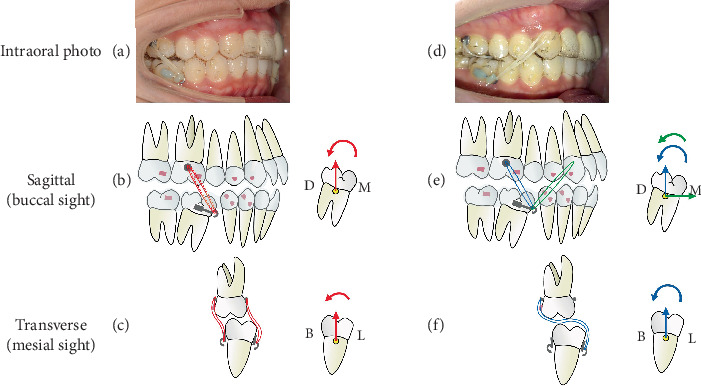
Mechanic schematics. (a) Intraoral photo of the #32 stage. (b) Sagittal analysis of the vertical tractions (red). (c) Transverse analysis of the vertical tractions. (d) Intraoral photo of the #38 stage. (e) Sagittal analysis of the interactive traction (blue) and Class II traction (green). (f) Transverse analysis of the interactive traction.

Although clear aligners were good at achieving the intrusion and distalization movement [23], posterior teeth mesial movement was hard to realize due to the insufficient grip of the soft material to hold on to the teeth. There were many ways to assist aligners in teeth mesial movement, such as miniscrews, power arms, and cantilevers [24]. In this case, during the mesial movement of mandibular molars, Tooth 46 inclined mesially (Figure 6), so we adjusted the force system immediately by using horizontal power arms and elastics. We replaced the buttons with power arms on both the buccal and lingual sides of Tooth 46 to correct the inclination by elastic with Tooth 16 (Tooth 46's buccal arm is connected to Tooth 16's buccal button, and Tooth 46's lingual arm is connected to Tooth 16's lingual button). Because of the thickness of the material of the aligner, Tooth 16 had no extrusion. Several weeks later, in the case of the lingual inclination of Tooth 46, we connected Tooth 46's lingual arm to Tooth 16's buccal button and applied Class II traction from Tooth 46 to Tooth 13 (Figure 18). After 4 months of traction, the result was satisfactory.

**Figure 19:**
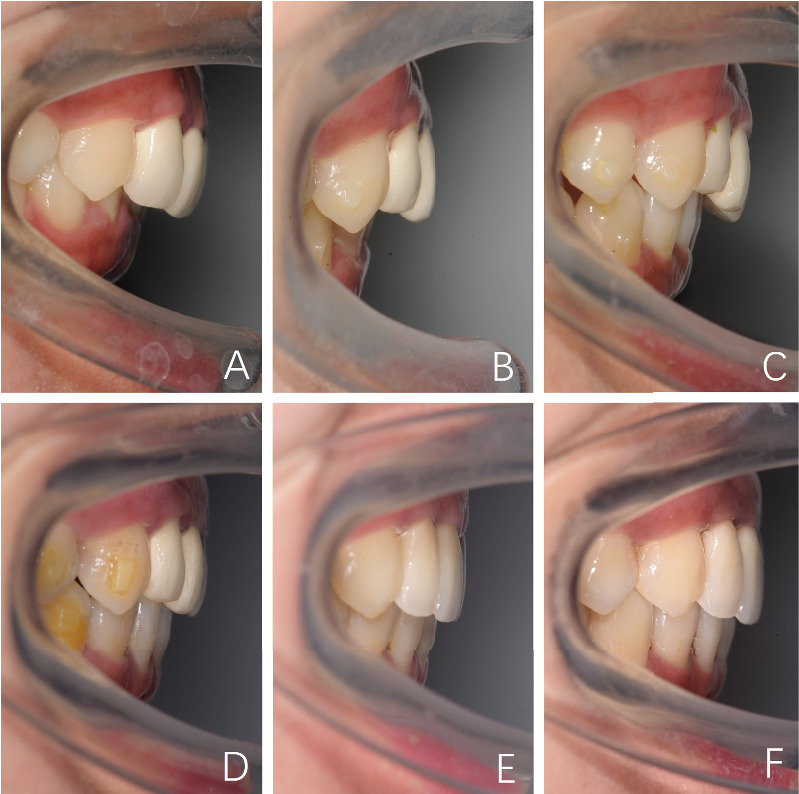
Progressive overjet views showing the overbite changes at (A) pretreatment, (B) the 8^th^ month, (C) the 12^th^ month, (D) the 24^th^ month, (E) posttreatment, and (F) retention after 15^th^ month.

After 12 months, a clear improvement was observed in the overbite and the axis of the upper incisors, indicating that clear aligners were good at correcting the deep bite (Figure 19). Unlike fixed appliances, clear aligners can utilize the material on the occlusion surface to intrude teeth efficiently [21]. At the end of the first phase, the space created by Tooth 45 extraction was used to correct the right molar relationship and to solve the lower arch crowding. After the first phase, the occlusion had been very nice. However, we designed a refinement phase with 15 steps of aligners in anticipation of maximum mandible counterclockwise rotation. As for improving the facial Class II profile, we designed to control the occlusion plane to make the mandible counterclockwise rotation by intruding the upper molar and the bimaxillary incisors in different degrees [25].

In orthodontic practice, unilateral extraction of a mandibular premolar is relatively less common than removal of a lower incisor. In this case, the compromised Tooth 45 was extracted, allowing Teeth 42–44 to functionally substitute for Teeth 43–45, which can be regarded as a single lower incisor extraction case. Although the right canine relationship was a full Class II, Teeth 13 and 42 (can be considered as the right lower canine) exhibited a Class I relationship. Interproximal enamel stripping was performed in the maxillary arch to optimize the normal range of the anterior Bolton ratio for Chinese. The maxillary midline was aligned with the midline of Tooth 31's crown, and normal overbite and overjet were achieved, resulting in a stable and functionally balanced occlusion.

**Figure 20:**
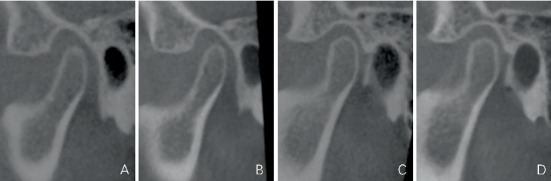
Sagittal view of TMJ on CBCT. (A) Pretreatment right TMJ. (B) Posttreatment right TMJ. (C) Pretreatment left TMJ. (D) Posttreatment left TMJ.

Class II Division 2 patients always suffered from TMD [26–28]. The position and morphology of the TMJ can be influenced by numerous factors, including deep bite and interincisal angle [29, 30]. A recent case report showed that TMD symptoms were alleviated after correcting anterior deep bite and leveling mandibular deep curves [31]. In this case, the labial inclination of the upper incisors can release the mandible, making the click during mouth opening disappear. Meanwhile, intrusion of the upper and lower incisors and the maxillary molar can control the occlusal plane, which can relocate the mandible into an accurate and stable place [32]. However, the sagittal view of the TMJ on CBCT shows no significant position difference between pretreatment and posttreatment (Figure 20). This may be because subtle changes in the articular disc are difficult to visualize in CBCT images. After orthodontic treatment, masticatory muscles can get a break when maximal occlusion is achieved, and correct anterior guidance can be established. Finally, the situation of the TMJ would get better. Class II traction was necessary for Class II patients' treatment, which cannot only help to avoid extra protrusion of the upper incisors but also make the TMJ adjust to a comfortable place. When treating the Class II Division 2 patients, we should add Class II traction after the upper incisors' axis is corrected completely. It is worth mentioning that if TMD symptoms aggravate during orthodontic treatment, intermaxillary traction should be immediately paused. In this case, by using Class II traction, the mandible was put in a stabilized position without destroying the morphology of the TMJ (Figure 20).

## 4. Conclusions

This case report represented a successful orthodontic treatment for a Class II Division 2 case with multiple porcelain fused to metal crowns and a severe deep bite. Cantilever arms and elastics were efficient in correcting mesial tipping during the mesial movement of the molar, which was relatively hard for clear aligners. Meanwhile, clear aligners were good at space control, simplifying the orthodontic treatment for patients with multiple crowns. The occlusal plane control allowed a counterclockwise rotation of the mandible to achieve facial esthetics and to alleviate TMJ symptoms.

## Figures and Tables

**Table 1:** Cephalometric measurements.

**Measurement**	**Standard norms**	**Pretreatment**	**Posttreatment**
*Bone*			
SNA (°)	83.0 ± 4.0	83.2	82.4
SNB (°)	80.0 ± 4.0	75.7	76.1
ANB (°)	3.0 ± 2.0	7.5	6.2
MP-SN (°)	33.0 ± 4.0	35.6	35.4
FH-MP(FMA) (°)	28.0 ± 4.0	26.4	26.0
S-Go/N-Me (%)	66.0 ± 4.0	63.9	63.6
*Teeth*			
U1-L1 (°)	127.0 ± 9.0	139.4	128.9
U1-SN (°)	105.7 ± 6.3	91.6	90.4
U1-NA (mm)	4.0 ± 2.0	0.1	1.0
U1-NA (°)	21.0 ± 6.0	8.4	8.1
L1-NB (mm)	6.0 ± 2.0	6.0	8.2
L1-NB (°)	28.0 ± 6.0	24.8	36.9
L1-FH (FMIA)	57.0 ± 7.0	60.1	48.7
L1-MP (IMPA)	109.0 ± 5.6	93.6	105.4
*Soft tissue*			
UL-EP (mm)	2.0 ± 2.0	2.1	1.6
LL-EP (mm)	3.0 ± 2.0	4.2	4.7
Z-angle (°)	71.0 ± 5.0	58.1	56.5

## Data Availability

The data that support the findings of this study are available from the corresponding author upon reasonable request.

## Nomenclature

TMD: temporomandibular disorder
TMJ: temporomandibular joint
PFM: porcelain fused to metal teeth
FMA: Frankfort mandibular plane angle
BSSRO: bilateral sagittal split ramus osteotomy
FMIA: Frankfort plane to mandibular incisor angle
CAT–CAT: clear aligner treatment complexity assessment tool
